# Spray-Type Adhesion Barrier Enhances Safety and Feasibility of Robotic Repeat Liver Resection: Initial Experience and Outcomes

**DOI:** 10.7759/cureus.59944

**Published:** 2024-05-09

**Authors:** Takahisa Fujikawa, Yusuke Uemoto, Kei Harada, Taisuke Matsuoka

**Affiliations:** 1 Surgery, Kokura Memorial Hospital, Kitakyushu, JPN

**Keywords:** postoperative complication, saline-linked cautery method, robotic liver resection, adhesiolysis, spray-type adhesion barrier

## Abstract

Background

Although various types of adhesion barriers are widely utilized in liver surgery, the safety and feasibility of their use during repeat robotic liver resection (R-RLR) are still unknown.

Methods

Among the 68 patients undergoing RLR with the application of the spray-type adhesion barrier at Kokura Memorial Hospital, Kitakyushu, Japan, between 2021 and 2023, 24 cases that underwent R-RLR were included in this study. The included patients were divided into two groups: those who underwent previous hepatectomy with the use of a spray-type adhesion barrier (R-RLR-B, n = 14) and those without its previous use (R-RLR-NB, n = 10). The perioperative outcomes were compared between the groups.

Results

There were no differences between the R-RLR-B and R-RLR-NB groups in background characteristics, difficulty scores, operative and console time, or surgical blood loss. Although no difference was found between the groups in the time required for adhesiolysis before the robotic operation, both the time required for robotic adhesiolysis (75 minutes vs. 58 minutes, p = 0.034) and total time for adhesiolysis (192 minutes vs. 141 minutes, p = 0.014) were significantly shorter in the R-RLR-B group than in the R-RLR-NB group. Otherwise, there was no conversion to open hepatectomy, no intraoperative transfusion of red blood cells, no cases of grade B or C post-hepatectomy liver failure, and no mortality in the whole cohort.

Conclusions

The spray-type adhesion barrier may not be associated with an increase in the incidence of postoperative complications, including bile leakage or intraperitoneal abscess. In addition, its application during the previous hepatectomy can facilitate a secure R-RLR with reduced time for adhesiolysis. Thus, the use of the spray-type adhesion barrier for R-RLR is safe, effective, and time efficient.

## Introduction

With the progress of aggressive multidisciplinary treatment for advanced cancer, the importance of repeat liver resection for intrahepatic recurrence of hepatocellular carcinoma (HCC) and colorectal cancer liver metastases (CRCLM) has been debated [1,2]. However, due to intraperitoneal adhesions that form after the first liver resection, not only the oncological curability but also the safety of the procedure may be compromised during a repeat liver resection. In recent years, several types of adhesion barriers have been developed that have the potential to reduce the risk of adhesion formation and associated surgical complications, and their use in various abdominal surgeries has increased. On the other hand, unlike other abdominal surgeries, since hepatectomy can cause unique postoperative complications associated with hepatic parenchymal resection, such as bile leakage and intractable ascites [3,4], risks and benefits must be carefully considered when using an adhesion barrier during liver resection.

Among the currently available adhesion barrier agents, a spray-type adhesion barrier (AdSpray™, Terumo Corporation, Tokyo, Japan) has gel-like properties based on N-hydroxysuccinimide-enriched carboxymethyl dextrin polymer [5], and the application of this spray-type adhesion barrier is considered suitable for the situation after resection of three-dimensional structures like the liver. A study using a rat hepatectomy model was the first to show that its use contributed to a reduction in intraabdominal adhesions [5]. Following extensive experience in a clinical setting, its safety and efficacy have been reported in patients undergoing open liver resection [6,7].

Robotic surgery has become widespread and is now being performed in various fields. Robotic liver resection (RLR) has the advantages of less blood loss, shorter recovery time, and fewer postoperative complications related to adhesion formation compared to conventional open surgery [8-10]. Although there have been some studies on the effectiveness and safety of the spray-type adhesion barrier in open repeat liver resection [6,7], there have been scarce studies on the effects of adhesion barriers in repeat RLR (R-RLR). In the current study, we evaluated the safety and efficacy of using the spray-type adhesion barrier in R-RLR.

## Materials and methods

This single-center retrospective cohort study included 68 patients undergoing RLR with the application of the spray-type adhesion barrier (Adspray™) at Kokura Memorial Hospital, Kitakyushu, Japan, between September 2021 and December 2023, among which 24 cases received R-RLR. Our exclusion criteria for RLR were patients with liver tumors 10 cm or larger in size or those with tumors being considered for vascular reconstruction or multivisceral resection; otherwise, all the hepatectomy procedures were scheduled to be performed as RLR. We introduced the utility of the spray-type adhesion barrier during liver resection in the middle of 2019, and it has been routinely used since then. All 24 patients undergoing R-RLR were divided into two groups: those who underwent previous hepatectomy with the use of the spray-type adhesion barrier (R-RLR-B, n = 14) and those undergoing previous hepatectomy without its previous use (R-RLR-NB, n = 10), which was regarded as a historical control. To assess the efficacy and safety of the spray-type adhesion barrier, we measured the time required for adhesiolysis before or during the robotic procedure, and the perioperative outcomes were compared between the groups.

Data on demographics or surgical procedures were obtained through a methodical analysis of healthcare records and a prospectively collected database. In assessing our procedure, we examined not only the surgical outcomes but also its practicability and safety. We used the IWATE criteria [11] on a scale from 0 to 12 to assess the level of difficulty of RLR. The Clavien-Dindo classification (CDC) [12] was employed to classify and assess postoperative complications; issues classified as CDC class IIIa or greater were considered to be of considerable importance. The occurrence of death within 30 days after a surgical procedure was denoted as operative mortality.

The research protocol was in accordance with the principles of ethics delineated in the Declaration of Helsinki and obtained approval (#21021002) from the Clinical Research Ethics Committee at Kokura Memorial Hospital.

Statistical analysis

Categorical data are typically presented in the form of absolute values and percentages, whereas continuous variables are denoted by the median and range. To compare continuous variables, the Mann-Whitney U-test was employed to compare continuous variables, while Fisher’s exact probability test was utilized to assess categorical values. Two-sided p-values <0.05 were regarded as statistically significant. Every analysis of statistics was performed utilizing the EZR (Saitama Medical Center, Saitama, Japan) [13], a graphical user interface for R (version 2.13.0, R Foundation, Vienna, Austria).

Surgical technique

All RLRs in the current series were performed using the Da Vinci Xi surgical system (Intuitive Surgical, Inc., Sunnyvale, California, USA). The patient position and port placement during RLR were previously described [14,15]. Briefly, in the case of the anterolateral (S2, S3, S4b, S5, and S6) or superior segments (S8 and S4a), the patient was positioned supine with their legs spread apart in a 10-degree reverse Trendelenburg position. On the other hand, in the case of the posterior segment, the patient was placed in a left lateral decubitus position, and the head side was raised 10 degrees.

A steady 8 mmHg was maintained as the intraabdominal pressure. Before parenchymal transection, the Pringle maneuver was prepared through the extraperitoneal tourniquet system in a standardized manner described in the previous study [16] and used on demand. We generally used monopolar curved scissors or the double bipolar method for dissection around the liver or adhesiolysis [17], whereas the saline-linked cautery (SLiC) method utilizing monopolar curved scissors or Maryland bipolar forceps combined with saline dripping was exclusively performed during liver parenchymal transection [14,15]. For the SLiC method, the assistant surgeon prepared to handle the ball-tipped SLiC linked to sterile 0.9% saline with a drip rate of 1-2 cc/minute and applied the saline droplets to the tip of the robotic cautery.

**Figure 1:**
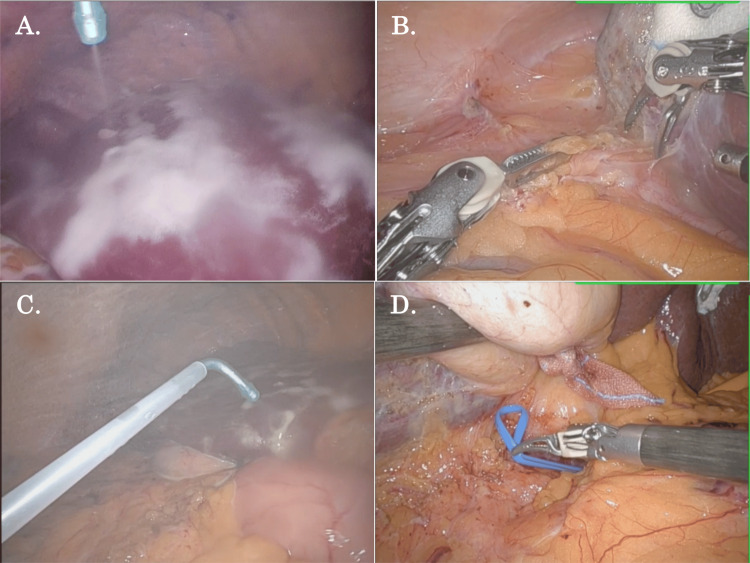
The process of robotic repeat liver resection after the previous laparoscopic liver resection with the use of a spray-type adhesion barrier (A) In the final step of the previous laparoscopic S6/7 partial liver resection, the spray-type adhesion barrier was applied around the hepatoduodenal ligament and liver surface widely and sparsely. (B) During the following robotic repeat S6 partial liver resection, the adhesion around the liver was subclinical and mild, which was easily adhesiolyzed by robotic dissection. (C) In the final step of the robotic repeat S6 partial liver resection, the spray-type adhesion barrier was also applied in the same fashion as the previous operation. (D) In the next robotic repeat S6 subsectionectomy of the liver, the adhesion was slightly expanded beneath the liver but could also be fully detached, and the preparation for Pringle’s maneuver could be achieved relatively easily.

A typical case of R-RLR with the previous use of the spray-type adhesion barrier is shown in Figure 1 and Video 1. In the final step of the previous laparoscopic S6/7 partial liver resection, the spray-type adhesion barrier was applied around the hepatoduodenal ligament and liver surface widely and sparsely (Figure 1A). During the following robotic repeat S6 partial liver resection, the adhesion around the liver was subclinical and mild, which was easily adhesiolyzed by robotic dissection (Figure 1B), and the spray-type adhesion barrier was also applied in the same fashion as the previous operation in the last step of the procedure (Figure 1C). In the next robotic repeat S6 subsectionectomy of the liver, the adhesion was slightly expanded beneath the liver but could also be fully detached, and the preparation for Pringle’s maneuver could be achieved relatively easily (Figure 1D).

**Video 1:** The process of robotic repeat liver resection after the previous laparoscopic liver resection with the use of a spray-type adhesion barrier

**Figure 2:**
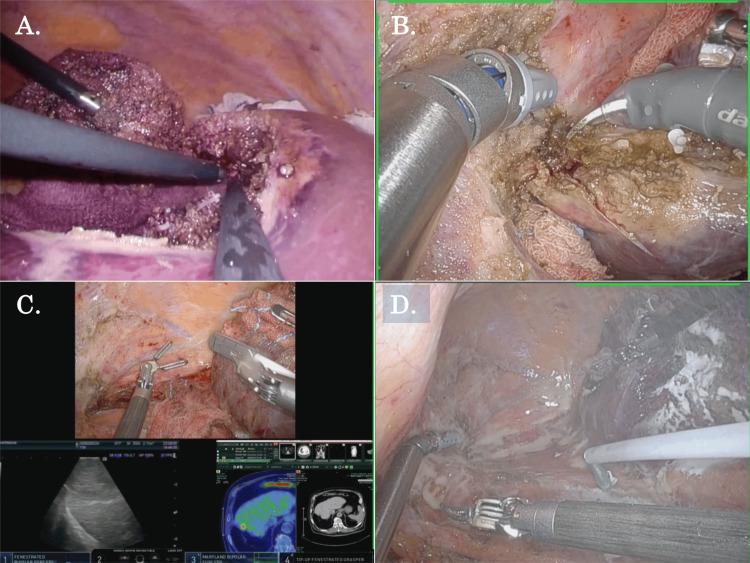
The process of robotic repeat liver resection after the previous laparoscopic liver resection without the use of an adhesion barrier (A) During the initial laparoscopic S6/7 partial liver resection, no adhesion barrier was applied. (B) During the following robotic repeat S7 partial liver resection, severe adhesion was widely formed around the liver. (C) Because very severe adhesions had formed, it took a lot of time to remove the adhesions. (D) After liver parenchymal transection, the spray-type adhesion barrier was applied widely around the liver.

On the other hand, Figure 2 and Video 2 outline the typical case of R-RLR without the previous use of adhesion barriers. During the initial laparoscopic S6/7 partial liver resection, no adhesion barrier was applied (Figure 2A). During the following robotic repeat S7 partial liver resection, severe adhesions were widely formed, especially in the area between the previous liver cut surface and the retroperitoneum (Figure 2B), and it took a lot of time to remove the adhesions (Figure 2C). After liver parenchymal transection and resection of the S7 tumor, the spray-type adhesion barrier was applied widely around the liver (Figure 2D).

**Video 2:** The process of robotic repeat liver resection after the previous laparoscopic liver resection without the use of an adhesion barrier

## Results

Among 68 patients who underwent RLR with the use of the spray-type adhesion barrier, including 23 patients (33.8%) who received preoperative chemotherapy, only one patient suffered from CDC IIIa bile leakage (1.5%), but no intraabdominal abscess was experienced. To assess the efficacy and safety of the spray-type adhesion barrier in R-RLR, we compared 14 patients in the R-RLR-B group to 10 cases in the R-RLR-NB group. The patient and tumor characteristics of the included cases are shown in Table 1. There were no differences in any background characteristics, including age, gender, liver functional status, type of disease, and size of the tumors.

**Table 1:** Patient and tumor characteristics in the current cohort CRCLM, colorectal cancer liver metastasis; HCC, hepatocellular carcinoma; ICC, intrahepatic cholangiocarcinoma; RLR, robotic liver resection; R-RLR, repeat robotic liver resection

Factors	R-RLR-NB (n = 10)	R-RLR-B (n = 14)	p-Value
Age, y, median (range)	76 (69-85)	75 (49-86)	0.577
Female sex, n (%)	4 (40.0)	4 (28.6)	0.673
Child-Pugh class B, n (%)	5 (50.0)	5 (35.7)	0.678
Disease: HCC, n (%)	7 (70.0)	5 (35.7)	0.214
Disease: CRCLM, n (%)	2 (20.0)	7 (50.0)	0.21
Disease: ICC, n (%)	1 (10.0)	2 (14.3)	1
Size of the tumor, mm, median (range)	20 (10-32)	24 (18-33)	0.308

**Figure 3:**
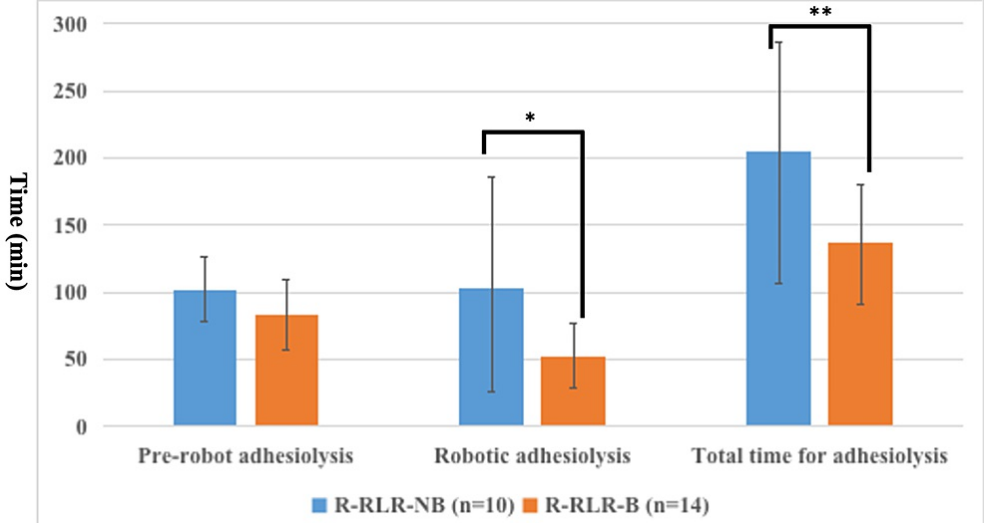
Assessment of time for adhesiolysis during R-RLR with reference to the previous use of a spray-type adhesion barrier Although no difference was found between the groups in the time required for adhesiolysis before robotic operation (pre-robot adhesiolysis), both the time required for robotic adhesiolysis (75 minutes vs. 58 minutes, p = 0.034)* and total time for adhesiolysis (192 minutes vs. 141 minutes, p = 0.014)** were significantly longer in the R-RLR-NB group than in the R-RLR-B group. R-RLR, repeat robotic liver resection

Table 2 displays the surgical factors and outcomes for the included patients. No differences were found between the groups in the difficulty scores, operative and console time, or surgical blood loss. A tendency toward a lower incidence of anatomical hepatectomy was observed in the historical control group (R-RLR-NB), mainly because more patients received previous anatomical hepatectomy in the R-RLR-NB group than in the R-RLR-B group. Although no difference was found between the groups in the time required for adhesiolysis before robotic operation (101 minutes vs. 81 minutes, p = 0.111), both the time required for robotic adhesiolysis (75 minutes vs. 58 minutes, p = 0.034) and total time for adhesiolysis (192 minutes vs. 141 minutes, p = 0.014) were significantly longer in the R-RLR-NB group than in the R-RLR-B group (Figure 3). Otherwise, there was no conversion to open hepatectomy, no intraoperative transfusion of red blood cells, no cases of grade B or C post-hepatectomy liver failure, and no mortality in the whole cohort. Only one postoperative complication with Clavien-Dindo class IIIa occurred (bile leak in 1) in the R-RLR-B group. The patient received preoperative chemotherapy and a subsequent robotic repeat S8 liver resection for recurrent CRCLM after the initial robotic S2 partial liver resection. He experienced postoperative bile leakage on day 5, but it improved conservatively. Otherwise, there were no specific postoperative complications related to the use of the spray-type adhesion barrier, such as intraperitoneal abscesses.

**Table 2:** Surgical factors and outcomes in the current cohort * Statistically significant CDC, Clavien-Dindo classification; LOS, length of postoperative stay; PHLF, post-hepatectomy liver failure; RLR, robotic liver resection; R-RLR, repeat robotic liver resection

Factors	R-RLR-NB (n = 10)	R-RLR-B (n = 14)	p-Value
Hepatectomy three or more times, n (%)	4 (40.0)	4 (28.6)	0.673
Previous operation: anatomical hepatectomy, n (%)	5 (50.0)	2 (14.3)	0.085
Current operation type: anatomical hepatectomy, n (%)	1 (10.0)	7 (50.0)	0.079
Difficulty score, median (range)	5 (2-10)	4 (1-10)	0.371
Operative time, min, median (range)	281 (179-622)	305 (90-535)	0.693
Console time, min, median (range)	180 (83-476)	228 (38-413)	0.921
Time for pre-robot adhesiolysis, median (range)	101 (70-146)	81 (52-153)	0.111
Time for robotic adhesiolysis, median (range)	75 (6-292)	58 (3-80)	0.036^*^
Total time for adhesiolysis, median (range)	192 (112-378)	141 (55-233)	0.014^*^
Blood loss, mL, median (range)	5 (5-60)	14 (5-60)	0.496
Red blood cell transfusion, n (%)	0 (0.0)	0 (0.0)	-
Conversion to open surgery, n (%)	0 (0.0)	0 (0.0)	-
PHLF (Grade B, C)	0 (0.0)	0 (0.0)	-
Mortality, n (%)	0 (0.0)	0 (0.0)	-
Postoperative. complication (CDC 3a or higher)	0 (0.0)	1 (7.7)	1
LOS, d, median (range)	7 (5-14)	7 (5-25)	0.473

## Discussion

In the current study, we evaluated the safety and efficacy of using a spray-type adhesion barrier in R-RLR. Although the background characteristics, difficulty scores, operative and console time, and surgical blood loss were similar, both the time required for robotic adhesiolysis and the total time for adhesiolysis were significantly shorter in the case of previous use of the spray-type adhesion barrier than those without its previous use. Moreover, there was no conversion to open hepatectomy, no mortality, and no increase in postoperative complications, including biliary leakage or intraabdominal abscess, in the whole cohort. Thus, the use of the spray-type adhesion barrier is safe, time efficient, and beneficial for R-RLR. To the best of our knowledge, this is the first study to assess both the safety and efficacy of the spray-type adhesion barrier for R-RLR.

Concerning the safety of using an adhesion barrier, several studies have looked into this topic. While previous research has not found a correlation between the use of adhesion barriers and increased morbidity after abdominal surgery, one study found an association between the wrapping of sheet-type adhesion barriers around surgical anastomosis in abdominopelvic surgery and an increased risk of anastomotic leakage and abscess formation [18]. Considering the potential contribution of postoperative adhesion formation to the early healing of wounds following surgical operations, one might anticipate that preventing adhesion formation could elevate the likelihood of significant morbidities. Hence, the utilization of adhesion barriers during abdominal surgery ought to be evaluated in light of the potential advantages and disadvantages for individual patients.

In the case of open liver resection, the safety of using the spray-type adhesion barrier has been reported after extensive experience in a clinical setting [6,7]. Conversely, a study documented that the solution state of the substance could potentially impede the adhesion required for healing at the cut surface of the liver. This could lead to a higher occurrence of intraabdominal abscesses following open liver surgery, particularly among patients undergoing preoperative chemotherapy [7]. To date, the safety of the spray-type adhesion barrier in liver surgery is still controversial, and the data on the safety of minimally invasive surgery, especially in RLR, is scarce. In the current study, among 68 patients who underwent RLR with the use of the spray-type adhesion barrier, including 23 patients (33.8%) who received preoperative chemotherapy, only one patient suffered from CDC IIIa bile leakage (1.5%), but no intraabdominal abscess was experienced. Moreover, the use of the spray-type adhesion barrier during R-RLR did not contribute to the increased incidence of biliary leakage or intraabdominal abscess. It is suggested that the spray-type adhesion barrier be safely used in RLR, regardless of whether preoperative chemotherapy was given or not or whether the status of hepatectomy was initial or repeated.

Regarding the adhesion barrier’s effectiveness, some studies conducted to date have shown that the application of adhesion barriers may lessen the severity of adhesions and the frequency of postoperative intestinal obstruction or potentially enhance the safety of repeat hepatectomy [19-23]. However, there is so far no definitive study assessing the efficacy of the spray-type adhesion barrier for R-RLR. In the current study, both the time required for robotic adhesiolysis and the total time for adhesiolysis were significantly shorter in the case of previous use of the spray-type adhesion barrier than those without its previous use. Furthermore, its use during R-RLR did not contribute to the increased incidence of adhesion-related complications, including postoperative ileus or bowel obstruction. Thus, it is suggested that the use of the spray-type adhesion barrier could potentially contribute to the safety of R-RLR.

Limitations of the study

The current study is subject to some limitations. Firstly, the study’s applicability in ascertaining the impact of treatments on outcomes is restricted due to its retrospective design. Secondly, the sample size appeared to be small; a larger sample size would probably yield suggestions that are more reliable. However, we firmly believe that this approach is a step toward the eventual standardization of RLR. It could result in better security and results and be another step toward the general application of a spray-type adhesion barrier during RLR that is safe and effective.

## Conclusions

Utilizing the spray-type adhesion barrier during R-RLR is safe and feasible. The use of this type of adhesion barrier may not be associated with an increase in the incidence of postoperative complications, including bile leakage or intraperitoneal abscess. In addition, its application during the previous hepatectomy can facilitate a secure R-RLR with reduced time for adhesiolysis.
